# Foetal cord blood contains higher portions of n-3 and n-6 long-chain PUFA but lower portions of *trans* C18:1 isomers than maternal blood

**DOI:** 10.3402/fnr.v59.29348

**Published:** 2015-11-27

**Authors:** Wiebke Schlörmann, Ronny Kramer, Alfred Lochner, Carsten Rohrer, Ekkehard Schleussner, Gerhard Jahreis, Katrin Kuhnt

**Affiliations:** 1 Department of Nutritional Physiology, Institute of Nutrition, Friedrich Schiller University Jena, Jena, Germany; 2 Placenta Laboratory, Department of Obstetrics, Jena University Hospital, Friedrich Schiller University Jena, Jena, Germany

**Keywords:** conjugated linoleic acids, pregnancy, *trans* fatty acids, elaidic acid, vaccenic acid, ruminant fat, omega-3 fatty acids

## Abstract

**Background/objective:**

An adequate supply of long-chain polyunsaturated fatty acids (LC PUFA) promotes foetal health and development, whereas generally, *trans* fatty acids (*t*FA) are considered to negatively interfere with LC PUFA metabolism. Nevertheless, to date, limited data concerning separate *trans* C18:1, such as *t*9 and *t*11, are available for maternal and foetal blood. Therefore, in this study the portions of individual *trans* C18:1, LC n-6, and n-3 PUFA in lipids of maternal and foetal plasma and erythrocyte membranes of German mother and child pairs (*n=*40) were analysed.

**Results:**

Portions of linoleic acid and α-linolenic acid as LC precursors were lower (~0.4-fold); whereas the metabolites arachidonic acid (AA, n-6) and docosahexaenoic acid (DHA, n-3) were significantly higher (~2-fold) in foetal than in maternal plasma and erythrocytes. The main *t*FA in maternal and foetal blood were elaidic acid (C18:1*t*9; *t*9) and vaccenic acid (C18:1*t*11; *t*11). Portions of *t*9, *t*10, *t*11, and *t*12 in foetal blood lipids were lower (~0.5-fold) compared with maternal blood. In foetal lipids, *t*9 was higher than *t*11. The *t*9 correlated negatively with eicosapentaenoic acid (n-3) and AA in maternal and foetal lipids; whereas *t*11 correlated negatively only with foetal total LC n-6 (plasma and erythrocytes) and n-3 PUFA (erythrocytes). No correlation between maternal *t*FA and foetal PUFA was observed.

**Conclusions:**

‘Biomagnification’ of LC n-6 and n-3 PUFA AA and DHA in foetal blood was confirmed, whereas single *trans* isomers were lower compared with maternal blood. Nevertheless, *t*FA intake, especially from industrial sources, should be as low as possible.

The essential dietary fatty acids (FA) linoleic acid (C18:2 n-6; LA) and α-linolenic acid (C18:3 n-3; ALA) are necessary for the endogenous synthesis of the long-chain polyunsaturated fatty acids (LC PUFA) arachidonic acid (C20:4 n-6; AA) and docosahexaenoic acid (C22:6 n-3; DHA) (1). These metabolites, particularly DHA, are important structural components, such as of cell membranes of the retina and the nervous system, suggesting an essential role for foetal and infant brain development (1). Studies also show a positive relationship between AA status and infant growth (2), as well as between the direct intake of LC omega-3 (n-3) PUFA and the length of gestation, birth weight, and a reduced risk of preterm delivery (3, 4). Because LA and ALA, as essential precursors of LC PUFA, cannot be synthesised *de novo*, the human foetus has to obtain these FA exclusively from the maternal diet by placental transfer. Foetal tissue is able to convert ALA into DHA and LA into AA as shown by Carnielli et al. (5) for premature infants, but the activity of elongation and desaturation enzymes is limited (6). Therefore, DHA and AA are predominantly derived by placental transfer from the mother in relation to the maternal FA metabolism and diet and accumulate in the foetus *in utero* (7, 8). It could be shown that DHA and AA are preferentially transferred to the foetus compared with other FA by the placenta (9, 10). This ‘biomagnification’ results in higher relative proportions of these LC PUFA in foetal blood lipids compared with maternal circulating lipids (11). Most of the DHA is stored in foetal adipose tissue, probably to ensure a constant supply of DHA for the first 2 months after birth (11, 12). Compared with the relatively stable AA levels, DHA in human breast milk is subjected to fluctuations depending on the maternal diet (13, 14). *Trans* fatty acids (*t*FA) in foetal tissue are exclusively derived from the maternal diet, perinatal by placental transport and postnatal by breast milk (7, 15). *t*FA are considered to exhibit adverse effects on essential FA and LC PUFA metabolism. The intake of essential FA can be reduced by the competition of *t*FA and essential FA or PUFA, respectively, which can result in negative effects regarding foetal and infant development (16). These negative effects are thought to be predominantly associated with a high intake of *t*FA from industrially produced foods such as hydrogenated vegetable oils, such as elaidic acid (C18:1 *t*9; *t*9), C18:1 *t*10 (*t*10), and *trans* C18:2 n-6, compared with *trans* isomers from natural sources such as dairy products, mainly vaccenic acid (C18:1 *t*11; *t*11) (17). In addition, ruminant-derived fat contains conjugated linoleic acids (CLA), which are isomers of LA with conjugated double bonds in *trans* and *cis* configurations. Studies show that ruminant-derived *t*11 and *c*9,*t*11 CLA in breast milk are associated with a lower risk of atopic outcomes in breast-fed children (18). In general, *t*11 and natural *c*9,*t*11 CLA exhibit beneficial health-related characteristics, such as anti-inflammatory effects as reviewed by Viladomiu et al. and Kuhnt et al. (19, 20).

The aim of the present study was to gain more insight into the LC PUFA and CLA status of foetal and maternal lipids as well as the profile of individual *trans* C18:1 isomers, such as *t*9, *t*10, *t*11, and *t*12. Therefore, the FA profiles including single *trans* C18:1 isomers were determined in maternal and foetal plasma and the respective erythrocyte membranes as well as in milk samples.

## Materials and methods

### Study subjects

The study protocol was approved by the ethics committee of the Friedrich Schiller University Jena and was part of the study no. 1354-06/04. Several cohorts were recruited in this study, for example, Enke et al. (21) referred to (2007–2008).

The present study was conducted from May to November 2005 prior to that of Enke et al. (21) who analysed a different study population, at the Jena University Hospital. Existing data from this earlier cohort were reanalysed to gain more insight into the distribution of individual *trans* isomers representing the different sources. In addition to the study of Enke et al. (21) breast milk samples of a subgroup of the cohort were also analysed.

Written informed consent was obtained from all subjects. The study population consisted of 40 mothers with their term-born infants (Table 1); however, breast milk samples could only be obtained from a subgroup of 20 mothers. All mothers and infants appeared healthy and there was no indication of diseases that could affect placental function.

**Table 1:** Characteristics of the mother and child pairs (*n*=40)

	Mean±SD
Age (years)	30.5±5.9
Week of gestation	38.9±1.4
Placental weight (g)	612±131
Birth length (cm)	50.2±2.1
Birth weight (g)	3,414±522

### Collection of maternal and cord blood, and breast milk samples

Blood from mothers was taken from the *vena basilica* at the time of birth and cord blood was obtained from the infants directly after its clamping. Blood was collected into the EDTA tubes. To separate erythrocytes and plasma, the blood was centrifuged at 1,500×*g* for 10 min at 8°C. In addition, breast milk (~15 ml) was obtained from a subgroup of mothers (*n*=20) sampled at 3–5 days postpartum [also called colostrum (22)]. Erythrocytes, plasma, and breast milk samples were stored at −80°C. FA distribution in all samples was analysed immediately after taking the samples of all the 40 mother/child pairs simultaneously (storage up to a maximum of 6 months).

### Preparation of erythrocyte membranes, plasma, and milk samples

After storage at −80°C, erythrocyte membranes were isolated by ultracentrifugation using the method of Burton et al. (23). All lipids were extracted with a chloroform/methanol/water mixture (1:1:0.9, v/v/v) according to Bligh and Dyer (24). Fatty acid methyl esters (FAME) from erythrocyte membranes were obtained using an acid-catalysed methylation with anhydrous HCl/methanol, as described by Dawczynski et al. (25). Lipids of plasma and breast milk samples were transformed to FAME by a combination of sodium methylate and 1,1,3,3-tetramethylguanidine (1,1,3,3-tetramethylguanidine in dry methanol, 1:4, v/v, 5 min, 100°C). All resulting FAME extracts were purified by thin-layer chromatography with hexane/diethyl ether/acetic acid mixture (26).

### Fatty acid analysis

FA analysis of purified FAME extracts was conducted via gas chromatography (GC, GC-17 V3; Shimadzu, Japan) equipped with an autosampler (AOC-5000) and a flame ionisation detector, as described by Kuhnt et al. (26). Two different GC procedures were performed using two different columns. A fused-silica capillary column DB-225ms (60 m×0.25 mm i.d., film thickness 0.25 µm; J&W Scientific, USA) was used to analyse FA ranging from 4 to 25 carbon atoms (including total CLA). A second high polar fused-silica capillary column CP select (200 m×0.25 mm i.d., film thickness 0.25 µm; Varian, the Netherlands) separated the *cis* and *trans* isomers of C18:1. Using this highly polar 200 m column, a good separation of *t*9, *t*10, *t*11, and *t*12 was achieved as shown with the 100 m CP-Sil 88 (27). The injector and detector temperatures were constant at 260 and 270°C, respectively, for both GC methods, using H_2_ as the carrier gas, as described by Kuhnt et al. (28). In all analysed materials, the same 55 FA were integrated including *trans* C18:1 isomers, using the GC solution software (Shimadzu). Individual FAME were expressed as a percentage of total identified FAME peak areas (% of total FAME).

### Statistical analysis

Statistical analysis of the data was performed using IBM SPSS statistics 19 (SPSS, Inc., Chicago, IL). Values are presented as mean with standard deviation (SD). *P*≤0.05 indicates significant difference. The Kolmogorov–Smirnov test was used to test the distribution of the data. Comparisons of two groups were performed with Student's *t*-test if the data were normally distributed. To calculate correlations, the Pearson correlation analysis was performed.

## Results

### Fatty acid profiles in maternal and foetal plasma and erythrocyte membranes

**Fig. 1:**
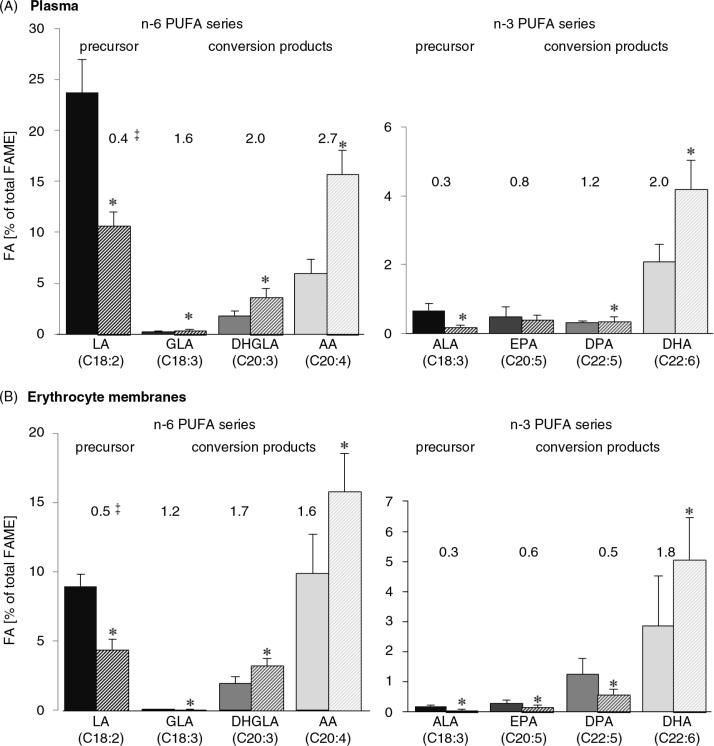
Portions of n-6 and n-3 PUFA precursors and long-chain metabolites in lipids of maternal (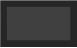; filled bars) and foetal (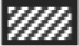; striped bars) plasma (A) and erythrocytes membranes (B). *Significantly different to the respective maternal FA (Student's *t*-test, *P*≤0.05). ^‡^Numbers indicate the ratio of foetal to maternal plasma portion of the respective fatty acid.

Regarding relative proportions of PUFA in total maternal and foetal blood lipids, AA was the major LC n-6 PUFA, whereas DHA was the major LC n-3 PUFA (Table 2). Levels of the precursors LA (n-6) and ALA (n-3) were significantly lower in foetal plasma and erythrocyte membranes (0.3- to 0.5-fold) compared with maternal plasma and erythrocytes (Table 2 and Fig. 1). In contrast, the respective LC metabolites such as γ-linolenic acid (GLA), dihomo-γ-linolenic acid (DHGLA), and AA (n-6), as well as docosapentaenoic acid (C22:5 n-3; DPA; only plasma) and DHA (n-3) showed up to 2.7-fold elevated levels in foetal plasma and erythrocyte membranes compared with maternal lipids (Table 2); except DPA and eicosapentaenoic acid (C20:5 n-3; EPA) in erythrocytes (Fig. 1). A significant correlation could be detected between maternal and foetal lipids for AA (plasma and erythrocytes) and for DHA (erythrocytes; Table 2).

**Table 2:** Fatty acid composition in maternal and foetal plasma and erythrocyte membranes

	Plasma (*n*=40)					Erythrocyte membranes (*n*=40)				
		
	Mean±SD			Correlation maternal vs. foetal		Mean±SD			Correlation maternal vs. foetal	
						
Fatty acids (% of total FAME)	Maternal	Foetal	*P*^a^	*r*	*P* ^b^	Maternal	Foetal	*P*^a^	*r*	*P*^b^
C14:0	1.09±0.31	0.75±0.16	***	−0.01		0.50±0.12	0.52±0.22		0.40	^##^
C15:0	0.27±0.05	0.20±0.04	***	0.07		0.24±0.06	0.18±0.04	***	0.52	^###^
C16:0	26.2±1.82	25.2±1.03	**	−0.26		26.4±3.02	28.17±2.82	***	0.48	^###^
C16:1 *c*9	3.43±1.11	4.51±0.94	***	0.24		0.64±0.19	0.77±0.15	***	0.22	
C17:0	0.25±0.03	0.27±0.10		0.09		0.39**±**0.09	0.33±0.06	***	0.57	^###^
C18:0	5.13±0.57	9.54±0.83	***	0.35	^#^	16.7±1.67	16.75±1.77		0.34	^#^
C18:1 *t*6–*t*8	0.03±0.01	0.01±0.01	***	0.63	^###^	0.03±0.02	0.01±0.01	***	−0.03	
C18:1 *t*9	0.15±0.03	0.08±0.02	***	0.46	^###^	0.12±0.03	0.07±0.02	***	0.20	
C18:1 *t*10	0.06±0.02	0.04±0.02	***	0.58	^###^	0.06±0.02	0.02±0.01	***	0.04	
C18:1 *t*11	0.09±0.03	0.05±0.02	***	0.14		0.14±0.03	0.05±0.02	***	0.24	
C18:1 *t*12	0.08±0.04	0.04±0.01	***	0.08		0.10±0.02	0.04±0.02	***	0.50	^###^
C18:1 *c*9	24.0±2.27	18.0±1.59	***	0.13		19.4±2.86	12.7±2.01	***	0.71	^###^
C18:1 *c*11	2.05±0.35	3.52±0.52	***	0.49	^###^	1.53±0.15	2.52±0.39	***	0.52	^###^
C18:1 *c*12	0.06±0.03	0.02±0.01	***	0.36	^#^	0.05±0.02	0.01±0.01	***	0.35	^#^
C18:1 *c*13	0.03±0.04	0.05±0.02	*	0.31		0.03±0.02	0.02±0.01	***	0.02	
C18:2 *c*9,*t*11 (CLA *c*9,*t*11)	0.26**±**0.06	0.23±0.06	***	0.42	^##^	0.09±0.03	0.06±0.02	***	0.50	^###^
C18:2 n-6 (LA)	23.7±3.22	10.6±1.33	***	0.16		8.95±0.87	4.39±0.75	***	0.07	
C18:3 n-6 (GLA)	0.24**±**0.11	0.39±0.07	***	−0.10		0.06±0.01	0.07±0.02	*	0.22	
C20:3 n-6 (DHGLA)	1.79±0.46	3.62±0.87	***	0.64	^###^	1.94±0.51	3.22±0.54	***	0.41	^##^
C20:4 n-6 (AA)	5.91±1.39	15.7±2.32	***	0.43	^##^	9.87±2.85	15.8±2.72	***	0.45	^###^
C22:4 n-6	0.00±0.01	0.00±0.01		0.13		0.44±0.31	0.28±0.49	*	0.33	^#^
C22:5 n-6	0.02±0.07	0.06±0.17		0.97	^###^	1.15±1.34	1.86±1.94	***	0.86	^###^
C18:3 n-3 (ALA)	0.67±0.20	0.18±0.05	***	−0.03		0.19±0.04	0.06±0.04	***	−0.23	
C20:5 n-3 (EPA)	0.47±0.29	0.38±0.14		0.03		0.28±0.12	0.17±0.07	***	0.29	
C22:5 n-3 (DPA)	0.30±0.06	0.35±0.12	*	−0.08		1.26±0.53	0.58±0.16	***	0.25	
C22:6 n-3 (DHA)	2.09±0.50	4.21±0.84	***	0.25		2.87±1.65	5.08±1.38	***	0.33	^#^
Σ SFAs	33.6±2.07	36.9±1.12	***	−0.18		48.9±4.71	50.7±4.05	*	0.42	^##^
Σ MUFAs	30.4±2.81	26.8±2.50	***	0.37	^#^	23.3±3.24	17.0±2.54	***	0.71	^###^
Σ PUFAs	36.1±4.03	36.5±2.82		0.19		27.9±6.02	32.2±4.63	***	0.43	^##^
Σ PUFAs n-3	3.60±0.77	5.26±1.02	***	0.19		4.66±2.25	5.94±1.56	***	0.31	^#^
Σ PUFAs n-6	29.0±3.94	22.8±8.40	***	0.60	^###^	23.1±4.04	26.2±3.44	***	0.42	^##^
Σ LC PUFAs n-3 (≥C_20_)	2.89±0.75	4.96±1.01	***	0.21		5.37±2.15	7.29±1.61	***	0.35	^#^
Σ LC PUFAs n-6 (≥C_20_)	13.9±1.86	27.5±2.58	***	0.36	^#^	20.7±5.62	30.8±4.49	***	0.46	^###^
Σ CLAs	0.30±0.08	0.31±0.13		0.46	^###^	0.18±0.09	0.13±0.07	***	0.49	^###^
Σ *trans* C18:1 (*t*6–*t*16)	0.53±0.14	0.47±0.13	*	0.23		0.60±0.12	0.41±0.08	***	0.46	^###^
*t*9/*t*11	1.75±0.43	2.04±0.84	*	0.03		0.90±0.24	1.44±0.53	***	0.07	

AA=arachidonic acid; ALA=alpha-linolenic acid; CLAs=conjugated linoleic acids; DHA=docosahexaenoic acid; DHGLA=dihomo-γ-linolenic acid; DPA=docosapentaenoic acid; EPA=eicosapentaenoic acid; FAME=fatty acid methyl esters; GLA=γ-linolenic acid; LA=linoleic acid; LC=long-chain; MUFA=monounsaturated fatty acids; PUFA=polyunsaturated fatty acids; SFA, saturated fatty acids.

a Significant differences between maternal and foetal plasma and erythrocytes fatty acids were calculated using Student's *t*-test (*P<0.05; **P<0.01; ***P<0.001);

b significant correlation between maternal and foetal plasma and erythrocytes fatty acids (*r*, Pearson's correlation coefficient; ^#^*P<*0.05; ^##^*P<*0.01; ^###^*P<*0.001).

**Fig. 2:**
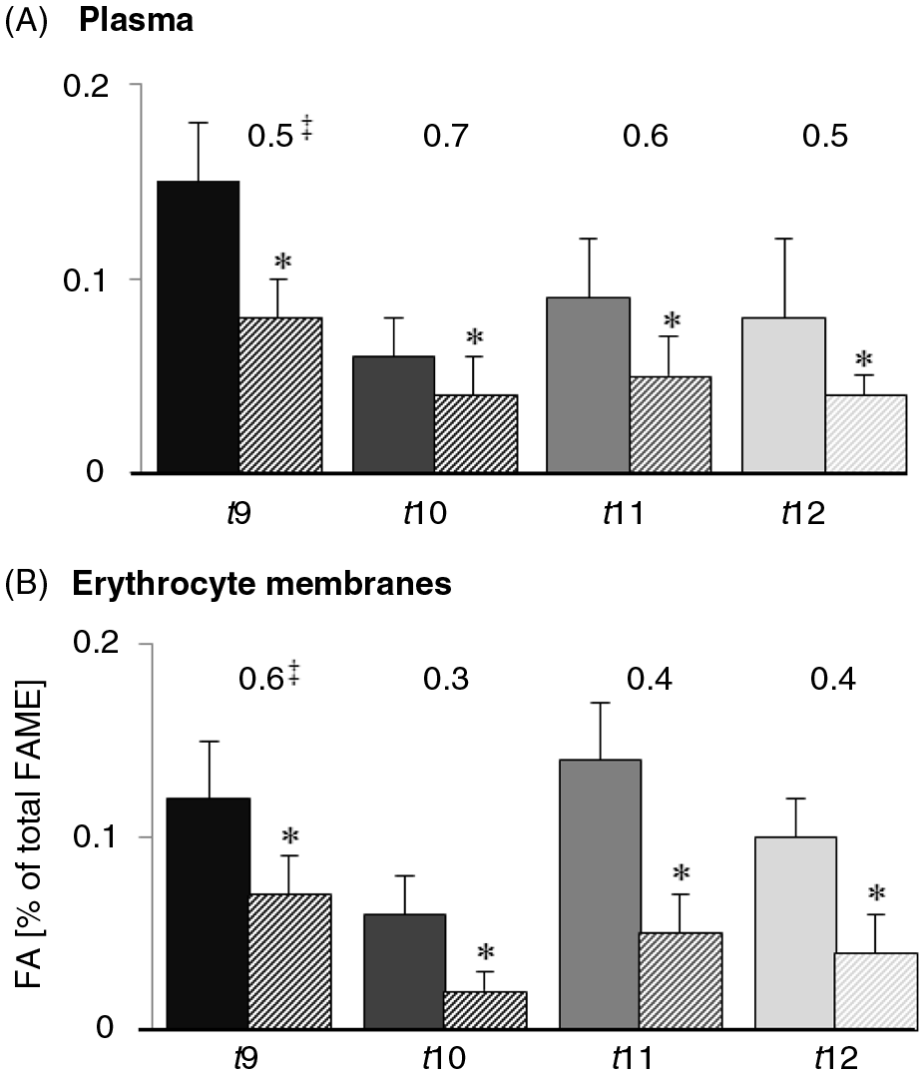
Portions of single *trans* C18:1 in lipids of maternal (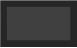; filled bars) and foetal (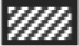; striped bars) plasma (A) and erythrocytes membranes (B). *Significantly different to the respective maternal FA (Student's *t*-test; *P*≤0.05). ^‡^Numbers indicate the ratio of foetal to maternal plasma portion of the respective fatty acid.

The major *trans* C18:1 isomers in maternal blood lipids were *t*9 and *t*11 (Table 2). Interestingly, the proportions of *trans* C18:1 isomers such as *t*9, *t*10, *t*11, and *t*12 were significantly lower in foetal than in maternal plasma (0.5- to 0.7-fold) as also in erythrocyte membranes (0.3- to 0.6-fold; Fig. 2). In foetal plasma and erythrocyte lipids, *t*11 was lower than *t*9, resulting in a higher foetal *t*9/*t*11 index compared with the maternal *t*9/*t*11 index. In plasma, *t*9 and *t*10 were significantly correlated between maternal and foetal lipids, but not *t*11 (Table 2).

The main CLA isomer *c*9,*t*11 CLA was generally higher in plasma compared with erythrocyte lipids. In addition, the maternal *c*9,*t*11 CLA portion was higher compared with the corresponding foetal lipids and showed a positive correlation. Generally, in maternal and foetal plasma, *c*9,*t*11 CLA portions were clearly higher compared with *t*11, but in erythrocytes both levels were fairly similar (Table 2).

### Correlation of *trans* C18:1 isomers with PUFA

Within maternal and foetal plasma and erythrocyte lipids, *t*9 was consistently inversely correlated with total LC n-3 PUFA and EPA as well as with LC n-6 PUFA and AA; whereas *t*10 only in maternal plasma. Negative correlations for *t*11 with total LC n-6 PUFA in plasma and erythrocytes and with total LC n-3 PUFA in plasma were exclusively detectable within foetal lipids, and for *t*12 only with EPA in maternal erythrocytes (Table 3).

**Table 3:** Correlation coefficients between single *trans* C18:1 isomers and LC PUFA in maternal and foetal lipids

	Plasma (*n*=40)						Erythrocyte membranes (*n*=40)					
		
	m/m		f/f		m/f		m/m		f/f		m/f	
						
	*r*	*P* a	*r*	*P* a	*r*	*P* b	*r*	*P* a	*r*	*P* a	*r*	*P* b
Σ *trans* C18:1 and Σ LC PUFA n-3 (≥C_20_)	−0.25		−0.12		−0.07		−0.40	**	−0.37	*	−0.15	
*t*9 and Σ LC PUFA n-3	−0.64	***	−0.38	*	−0.20		−0.51	***	−0.45	**	0.01	
*t*10 and Σ LC PUFA n-3	−0.55	***	−0.21		−0.12		−0.31		−0.24		−0.04	
*t*11 and Σ LC PUFA n-3	−0.05		0.02		0.02		−0.22		−0.43	**	−0.12	
*t*12 and Σ LC PUFA n-3	0.06		0.03		−0.12		−0.30		−0.16		0.01	
*t*9 and C20:5 n-3 (EPA)	−0.53	***	−0.42	**	−0.15		−0.42	**	−0.49	***	−0.03	
*t*10 and C20:5 n-3 (EPA)	−0.53	***	−0.34	*	−0.16		−0.32	*	−0.11		−0.17	
*t*11 and C20:5 n-3 (EPA)	0.08		−0.15		−0.26		−0.16		−0.21		−0.25	
*t*12 and C20:5 n-3 (EPA)	0.26		−0.12		−0.17		−0.33	*	0.14		−0.27	
Σ *trans* C18:1 and Σ LC PUFA n-6 (≥C_20_)	−0.45	**	−0.44	**	−0.19		−0.36	*	−0.46	**	−0.21	
*t*9 and Σ LC PUFA n-6	−0.58	***	−0.37	*	−0.15		−0.50	***	−0.60	***	−0.04	
*t*10 and Σ LC PUFA n-6	−0.61	***	−0.49	***	−0.24		−0.28		−0.31		−0.05	
*t*11 and Σ LC PUFA n-6	−0.30		−0.51	***	−0.08		−0.20		−0.43	**	−0.10	
*t*12 and Σ LC PUFA n-6	−0.30		−0.29		0.05		−0.26		−0.26		−0.06	
*t*9 and C20:4 n-6 (AA)	−0.63	***	−0.31	*	−0.16		−0.54	***	−0.50	***	−0.11	
*t*10 and C20:4 n-6 (AA)	−0.51	***	−0.26		−0.22		−0.26		−0.44	**	−0.03	
*t*11 and C20:4 n-6 (AA)	−0.24		−0.16		−0.19		−0.20		−0.17		−0.17	
*t*12 and C20:4 n-6 (AA)	−0.27		−0.15		−0.16		−0.19		−0.01		0.04	
Σ *trans* C18:1 and Σ CLA	0.37	*	0.20		0.59	^###^	0.25		0.15		−0.20	
*t*9 and *c*9,*t*11 CLA	0.22		0.47	**	0.41	^##^	−0.05		0.11		−0.20	
*t*11 and *c*9,*t*11 CLA	0.43	**	0.11		0.44	^##^	0.35	*	0.19		0.18	

AA=arachidonic acid; CLA=conjugated linoleic acids; EPA=eicosapentaenoic acid; f=foetal; LC=long-chain (≥C_20_); m=maternal; PUFA=polyunsaturated fatty acids; *r=*Pearson's correlation coefficient.

a Within FA of maternal or foetal plasma and erythrocyte fatty acids (*P<0.05, **P<0.01, ***P<0.001);

b between maternal and foetal blood lipids (^#^*P<*0.05, ^##^ *P<*0.01, ^###^*P<*0.001).

However, there was no correlation between maternal *trans* C18:1 isomers and foetal individual LC n-3 and n-6 PUFA. In contrast, maternal plasma *t*9 and *t*11 isomers correlated positively with foetal plasma *c*9,*t*11 CLA (Table 3).

### *trans* Fatty acid profile in breast milk lipids

The total *trans* C18:1 portion in breast milk samples (*n*=20) was 1.4%, and the portions of individual *t*9, *t*10, *t*11, and *t*12 isomers were 0.35% (0.08), 0.22% (0.05), 0.28% (0.07), and 0.18% (0.03), respectively, which were higher compared with portions in maternal and foetal plasma and erythrocytes (*P*≤0.05). The *t*9/*t*11 index reflected the continuously higher portion of *t*9 versus *t*11, with the lowest difference in breast milk (1.4), followed by maternal (1.7) and foetal plasma lipids (2.1). The *c*9,*t*11 CLA portion in breast milk was with 0.28% (0.04) similar to the *t*11 portion. A positive correlation was found between *t*11 and *c*9,*t*11 CLA (*r*=0.57, *P*≤0.01), but not between *t*9 and *c*9,*t*11 CLA (*r*=0.03). Total *trans* C18:1 in maternal plasma was solely correlated with EPA in breast milk (*r=*0.48, *P*≤0.05). Here, when separated into single *trans* C18:1, only *t*11 was significantly positively correlated with breast milk EPA (*r*=*t*9: 0.19, NS; *t*10: 0.13, NS; *t*11: 0.48, *P*≤0.05; *t*12=0.41, NS).

## Discussion

DHA and AA are important for foetal growth and development. They can be transferred to the foetus primarily via placental transfer or breastfeeding after birth (28, 29). Although AA and DHA can also be synthesised from precursors in foetal tissue, the foetal availability of these FA, especially DHA, depends on the maternal diet.

The concentration of maternal plasma triglycerides elevates progressively through pregnancy (29). Compared with the foetus, the mother has a higher absolute concentration of all plasma FA, including DHA and AA; however, the relative proportion of DHA and AA in FA of total circulating lipids, as we analysed, is consistently higher in the neonate (11). This was called ‘biomagnification’ (30). In the present study, it was clearly shown that the precursor portions of LA and ALA were higher in maternal compared with foetal plasma, whereas their LC metabolites, especially the final products such as AA and DHA, were elevated in foetal plasma. Similar results were found in lipids of isolated cell membranes of erythrocytes [consisting of 85% phospholipids (31)]. The measurement of FA in erythrocytes, however, may best reflect a long-term accumulation of FA indicating dietary intake and endogenous metabolism (31). In addition, lower precursor/product ratios (e.g. LA/AA, ALA/DHA) were found in foetal lipids in both plasma and erythrocyte membranes. These recent results confirmed the ‘biomagnification’ process, resulting from a preferential transfer of AA and DHA to the foetus (11, 30).

Moreover, foetal tissue is able to synthesise AA with an enhanced capacity compared with DHA (11) as indicated due to higher foetal plasma portion of AA compared with DHA in relation to maternal plasma (2.7-fold vs. 2.0-fold, respectively). Positive correlations between maternal and foetal PUFA in blood lipids were found for AA, DHA, DPA, and for total LC n-3 and n-6 PUFA (not consistent for plasma and erythrocytes). Similar results were obtained by Elias and Innis (7) and Enke et al. (21).

In contrast, *t*FA are not synthesised in foetal tissue but are exclusively transported by placental transfer or postnatal by breastfeeding (16). Therefore, foetal blood *t*FA originate from a maternal diet containing industrial hydrogenated vegetable oils (mainly *t*9 and *t*10) and ruminant fat (mainly *t*11) (7, 16, 32). Several studies suggest that *t*FA interfere with LC PUFA metabolism and, thereby, compromise foetal development (16, 32), whereas only a few studies distinguish between single *trans* isomers. For example, high concentration of *t*9 in maternal phospholipids was associated with lower birth weight in Dutch pregnant women (33). In another study from the Netherlands, lower birth weight and head circumference were significantly and negatively related to the *t*9 concentration in cord lipids (34), whereas *t*11 data were not available.

The present total *trans* C18:1 levels in maternal plasma lipids were higher compared with foetal plasma lipids, ranging from 0.47 to 0.60% FAME. These results can be associated with low maternal *t*FA intake (<1% of energy intake, En%) (35, 36), which is also supported by data presented by the Federal Institute of Risk Assessment Germany who estimated that the *t*FA intake in Germany (2005–2007) was below 1 En% as reviewed by Kuhnt et al. (20). The results of the present study are in line with other studies that revealed higher proportions of *t*FA in maternal lipid fractions (21, 37, 38). The portions of individual *trans* C18:1 isomers (*t*9, *t*10, *t*11, and *t*12) were significantly lower in foetal plasma compared with maternal plasma (~0.5-fold), confirmed by lower portion in the membrane lipids of erythrocytes (Fig. 2). Enke et al. (21) detected comparable portions of single *trans* C18:1 isomers in maternal and foetal lipids, also with generally lower portions in foetal lipids (~0.5-fold). The *t*11 in maternal lipids increased in relation to a high dairy fat consumption, but without increasing *t*11 in foetal lipids (21). Taken together, this indicates a reduced transfer of *trans* C18:1 to the foetus. Additionally, *t*FA might be located in an unfavourable position in triglycerides (*sn*-1 and *sn*-3) to be hydrolysed and released by placental lipoprotein lipase, which predominantly hydrolyses FA in *sn*-2 position as also discussed by Müller et al. for CLA (37). In the present study, *t*9 and *t*10 correlated positively between maternal and foetal plasma, whereas *t*11 showed no association (Table 2). On the contrary, Enke et al. (21) showed that independent of dairy consumption, maternal *t*11 was highly correlated with foetal *t*11, whereas *t*9 was not.

In dairy products, *c*9,*t*11 CLA, as the major CLA, originates mainly from the Δ9-desaturation of *t*11 (39). Studies showed that dairy fat intake correlated with CLA concentrations in plasma (21), adipose tissue (40), and breast milk (36). The present study revealed higher levels of *c*9,*t*11 CLA compared with its precursor *t*11 in plasma fractions, which could have resulted from the preferential conversion of *t*11 to *c*9,*t*11 CLA (26). The possibility of Δ9-desaturation distinguishes the *t*11 from the *t*9, *t*10, and *t*12 isomers (26). In erythrocytes, *c*9,*t*11 CLA was generally lower than in plasma lipids. Elias and Innis (7) showed that CLA could cross the human placenta and could be transferred to the foetus. Also, Müller et al. (37) reported a linear relationship between CLA in maternal and foetal blood lipids, as found in the present study, indicating placental CLA transfer. The natural CLA derived from dairy products may exhibit beneficial effects on foetal and infant health (16–18, 41).

*t*FA may interfere with LC PUFA metabolism, for example, by blocking the transfer of PUFA to the foetus or by inhibiting the conversion of precursors into LC PUFA (7, 16, 42, 43). The influence of individual *t*FA on LC PUFA status is not sufficiently analysed. In the present study, only *t*9 correlated consistently inversely with EPA and AA within both maternal and foetal lipids, whereas *t*10 only in maternal plasma (Table 3). Similar correlation trends were reported (21). In a recent study, *t*9 was also inversely associated with LC PUFA, such as AA and DHA in the erythrocytes of pregnant women and in umbilical lipids; unfortunately, *t*11 was not analysed (44). In a recent study, Chisaguano et al. (45) showed that *t*9 was negatively and *t*11 positively correlated to n-3/n-6 PUFA, whereas *c*9,*t*11 correlated positively with n-3 PUFA in maternal plasma. However, the present work and that by Enke et al. (21) found no significant correlation between maternal *trans* C18:1 and foetal n-6 and n-3 PUFA, which strengthens the assumption that the relatively low maternal *t*FA levels did not directly influence the LC PUFA levels of foetal lipids.

FA composition of breast milk is influenced by a variety of variables (e.g. maternal nutritional status, dietary intake, and length of lactation) (22). *t*FA (46) and CLA (35) in human milk are elevated by maternal intake of these FA. The analysed breast milk lipids contained 1.4% *trans* C18:1, which can be associated with low maternal *t*FA intake (<1% of energy intake) (35, 36). In concordance with reducing the content of industrial *t*FA in foods, the *t*FA content in breast milk also decreased (46). The relatively high *t*9/*t*11 index and the moderate *c*9,*t*11 CLA portion in the breast milk samples indicate a maternal diet relatively low in dairy fat, as previously estimated (41).

In conclusion, the present data confirm higher portions of LC n-3 and n-6 metabolites, whereas portions of individual *trans* C18:1 isomers such as *t*9, *t*10, *t*11, and *t*12 were lower in foetal plasma and erythrocyte membranes compared with maternal blood lipids. The generally low *trans* C18:1 portions in this German cohort indicate weak influence on foetal PUFA status. However, it is generally recommended that *t*FA intake, especially of industrially derived *t*FA, should be as low as possible. Further research is necessary to elucidate more precisely the role of single *t*FA isomers in maternal and foetal metabolism, with special regard to industrial and ruminant origins.
